# 5-Methylindole Potentiates Aminoglycoside Against Gram-Positive Bacteria Including *Staphylococcus aureus* Persisters Under Hypoionic Conditions

**DOI:** 10.3389/fcimb.2020.00084

**Published:** 2020-02-28

**Authors:** Fengqi Sun, Mengmeng Bian, Zhongyan Li, Boyan Lv, Yuanyuan Gao, Yan Wang, Xinmiao Fu

**Affiliations:** ^1^Provincial University Key Laboratory of Cellular Stress Response and Metabolic Regulation, College of Life Sciences, Fujian Normal University, Fuzhou, China; ^2^Engineering Research Center of Industrial Microbiology of Ministry of Education, College of Life Sciences, Fujian Normal University, Fuzhou, China

**Keywords:** 5-Methylindole, indole, aminoglycoside, antibiotic resistance, antibiotic tolerance, persister, gram-positive bacteria, *Staphylococcus aureus*

## Abstract

Antibiotic resistance/tolerance has become a severe threat to human and animal health. To combat antibiotic-resistant/tolerant bacteria, it is of significance to improve the efficacy of traditional antibiotics. Here we show that indole potentiates tobramycin to kill stationary-phase *Staphylococcus aureus* cells after a short, combined treatment, with its derivative 5-methylindole being the most potent compound tested and with the absence of ions as a prerequisite. Consistently, this combined treatment also kills various types of *S. aureus* persister cells as induced by the protonophore CCCP, nutrient shift, or starvation, as well as methicillin-resistant *S. aureus* (MRSA) cells. Importantly, 5-methylindole potentiates tobramycin killing of *S. aureus* persisters in a mouse acute skin wound model. Furthermore, 5-methylindole facilitates killing of many strains of gram-positive pathogens such as *Staphylococcus epidermidis, Enterococcus faecalis*, and *Streptococcus pyogenes* by aminoglycoside antibiotics, whereas it suppresses the action of aminoglycoside against the gram-negative pathogens *Escherichia coli* and *Shigella flexneri*. In conclusion, our work may pave the way for the development of indole derivatives as adjuvants to potentiate aminoglycosides against gram-positive pathogens.

## Introduction

Antibiotic resistance/tolerance has become a severe threat to public health and global economic development (Lewis, 2010; WHO, 2014; O'Neill, 2016; Balaban et al., 2019). Currently, no single or simple strategy suffices to fully contain the emergence and spread of antibiotic-resistant/tolerant pathogens. The discovery and development of new antibiotics have played a dominant role in these attempts. Nevertheless, the new types of antibiotics brought into clinical application since the 1990s are very limited (WHO, 2014; Hoagland et al., 2016), presumably due to both scientific and financial barriers. Besides developing new antibiotics, improving the efficacy of traditional antibiotics is an important strategy for combating antibiotic-resistant/tolerant pathogens (WHO, 2014; Trusts, 2016). Accordingly, advantages of this strategy include the good documentation of the toxicity, pharmacokinetics, administration, and mechanisms of action of traditional antibiotics.

In the last decade, many adjuvants have been reported to enhance the action of existing antibiotics. For instance, iron chelators (Moreau-Marquis et al., 2009), plant steroid tomatidine (Mitchell et al., 2012), glycerol monolaurate and lauric acid (Hess et al., 2014), resveratrol (Nohr-Meldgaard et al., 2018), and β-lactam aztreonam (Yu et al., 2012) were found to enhance the aminoglycoside tobramycin against different pathogens. Further, various metabolites, such as glucose and alanine, were found to facilitate aminoglycoside antibiotics to eradicate different pathogenic persister cells by enhancing proton motive force (PMF)-dependent aminoglycoside uptake (Allison et al., 2011; Barraud et al., 2013; Peng et al., 2015; Meylan et al., 2017; Su et al., 2018). Notably, rhamnolipids, synthesized by *Pseudomonas aeruginosa*, potentiate aminoglycoside against *Staphylococcus aureus* persisters by enhancing aminoglycoside uptake in a PMF-independent manner (Radlinski et al., 2019).

Indole, an interkingdom signaling molecule, has important biological functions in bacteria and animals (Lee and Lee, 2010). For instance, it has been shown to increase the antibiotic tolerance of *Escherichia coli* cells (Lee et al., 2010; Han et al., 2011; Vega et al., 2013), likely by activating stress response-related pathways (Vega et al., 2012) and/or upregulating the expression of antibiotic-efflux pump genes (Hirakawa et al., 2005). Conversely, several studies have revealed that indole is able to reduce the antibiotic tolerance of *E. coli* (Hu et al., 2015; Kwan et al., 2015) and *Lysobacter enzymogenes* (Han et al., 2017; Wang et al., 2019), highlighting its potential as an adjuvant for antibiotic potentiation. In particular, halogenated indoles are even able to directly eradicate bacterial persister cells and biofilms (Lee et al., 2016). These studies suggest that indole may exert diverse effects under antibiotic challenge conditions in the context of growth conditions and cell status.

We recently reported that hypoionic shock (i.e., shock with an ion-free solution) could markedly facilitate aminoglycosides to kill stationary-phase *E. coli* cells, but exhibited limited effects against stationary-phase *S. aureus* cells (Jiafeng et al., 2015) and triggered *S. aureus* persister cells (Chen et al., 2019). We sought to enhance the efficacy of this unique approach against *S. aureus* persister cells. Here we found that not only *S. aureus* but also several gram-positive pathogens could be killed by a short, combined treatment using aminoglycoside and indole derivatives, with 5-methylindole (5M-indole) being the most potent. Our study may open an avenue to develop new strategies against gram-positive pathogens.

## Materials and Methods

### Bacterial Strains, Medium, and Reagents

Various gram-positive and gram-negative bacterial strains were used in this study (refer to Table S1). Over-night culture of each strain was diluted at 1:500 in Luria-Bertani (LB) medium (Note: MRS medium was used for *L. lactis* and *E. faecalis*) and agitated in a shaker (37°C, 220 rpm) for 3–6 and 20–24 h to prepare exponential-phase and stationary-phase cells, respectively. Aminoglycoside antibiotics are described in Table S2. Carbonyl cyanide m-chlorophenylhydrazone (CCCP) was purchased from Sigma-Aldrich. All other chemical reagents are of analytical purity. Indole, 2M-indole, 5M-indole and paraben, as dissolved in DMSO solution, were stocked in brown, opaque Eppendorf tubes to avoid photo-induced damage.

### Aminoglycoside Potentiation by Indole Under Hypoionic Condition

Briefly, 100 μL cell cultures were centrifuged (12, 000, *g*, 1 min) in Eppendorf tube and the supernatant was completely removed. Cell pellets were re-suspended and thoroughly mixed with the working solution, which was prepared by dissolving aminoglycoside antibiotic plus indole, 2M-indole, 5M-indole or paraben in pure water at concentrations as described in Table S2. Cell suspension was kept at room temperature for 5 min before washing twice with phosphate-buffered saline (PBS: 0.27 g/L KH_2_PO_4_, 1.42 g/L Na_2_HPO_4_, 8 g/L NaCl, 0.2 g/L KCl, pH 7.4), and then 4 μL of 10-fold serially diluted cell suspension were spot plated onto LB agar dishes for cell survival assay. Cycled combined treatments were performed by washing the treated cells with PBS once and then subjected to another round of treatment. The effect of salts and EDTA was examined by re-suspending the cell pellets with the working solution containing NaCl, KCl, MgCl_2_, BaCl_2_, CaCl_2_, or EDTA at various concentrations.

### Preparation and Eradication of Antibiotic-Tolerant *S. aureus* Persister Cells

ATP depletion-associated persisters were prepared by agitating *S. aureus* stationary-phase cell culture in the presence of 100 μM protonophore CCCP or 15 μM NaN_3_ for 1 h, and cells were then treated with the working solution containing CCCP or NaN_3_ at corresponding concentrations. Nutrient shift-induced persisters were prepared as previously reported (Radzikowski et al., 2016; Chen et al., 2019). Starvation-induced persisters were prepared as previously reported (Eng et al., 1991; Chen et al., 2019). Tobramycin-tolerant persister cells were prepared by adding tobramycin at a final concentration of 500 μg/mL into *S. aureus* stationary-phase cell culture and further agitating for 1 h prior to the combined treatment.

### Intracellular ATP Level Assay

A luciferase-based kit (BacTiter-Glo™ Microbial Cell Viability Assay, Promega Corporation, USA; Cat.# G8093) was used to measure ATP level according to the manufacturer's instruction. Briefly, *S. aureus* persister cells, with or without pretreatment of 100 μM CCCP or 15 μM NaN_3_ for 1 h, was lysed using the lysis buffer and centrifuged (12, 000, *g*, 4°C, 5 min). The supernatant was quickly mixed with the working solution at equal volumes and then transferred into a 96-well plate before light recording on a FLUOstar Omega Microplate Reader using the Luminometer method.

### Animal Experiments

A skin acute wound model was applied to test the *in vivo* efficacy of the combined treatment by referring to an earlier report (Davidson, 1998). Briefly, 8-week-old ICR male mice (around 28 g) were purchased from the Animal Center of Fujian Medical University and maintained in the Animal Center of Fujian Normal University. Mice were housed for 1 or 2 days and then randomly divided into four groups for surgery experiments (Group A: treatment with 0.9% NaCl solution; Group B: treatment with tobramycin in 0.9% NaCl solution; Group C: treatment with tobramycin in pure water; Group D: treatment with tobramycin and 5M-indole in pure water; *n* = 3). Mice were anesthetized by intraperitoneal injection of 4% chloral hydrate, barbered on the right back and sterilized, and then a 1 cm × 1 cm whole skin section was removed to make an acute skin wound. Five microliter of 10-fold-concentrated stationary-phase *S. aureus* cells were seeded on the wound and fully absorbed before adding 120 μL working solution (4 mM 5M-indole plus 100 μg/mL tobramycin) and further incubating for 5 min. Residue solution was removed by absorbing with medical cotton and then subjected to another round of the combined treatment. The whole muscle on the wound site was removed and homogenized, with the lysates being spot-plated on LB agar dishes for bacterial survival assay.

### Ethics

The animal use protocol was approved by the Animal Ethical and Welfare Committee of Fujian Normal University (approval No.: IACUC 20190006) and performed in accordance with the U.K. Animals (Scientific Procedures) Act, 1986 and associated guidelines, EU Directive 2010/63/EU for animal experiments, as well as with the National Standards of the People's Republic of China (GB/T 35892-2018: Laboratory animals-Guideline for ethical review of animal welfare; GB/T 35823-2018: Laboratory animals-General requirements for animal experiment).

### Statistics

CFU (colony-forming units) on LB agar dishes were counted and cell density was calculated according to the dilution fold and volume of cell suspension droplet. Quantitative data, as calculated by Microsoft Excel, represent means ± SD of three replicates from one independent experiment; independent experiments were repeated at least three times. A statistical analysis was performed in the MicroOrigin software using the ANOVA algorithm at a significance level of 0.05.

## Results

### Respective Potentiating and Suppressive Effects of 5M-Indole on Tobramycin Against Stationary- and Exponential-Phase *S. aureus* Cells Under Hypoionic Conditions

We first confirmed that the aminoglycoside antibiotics tobramycin, gentamicin, and kanamycin, when dissolved in pure water, but not in NaCl containing solution, were all able to effectively and rapidly (within a few minutes) kill stationary-phase *E. coli* cells (Figure S1A) but only had a limited lethal effect on stationary-phase *S. aureus* cells (Figure S1B). We sought to improve the efficacy of hypoionic shock-induced aminoglycoside potentiation by adding paraben, indole, 2-methylindole (2M-indole), or 5-methylindole (5M-indole).

**Figure 1:**
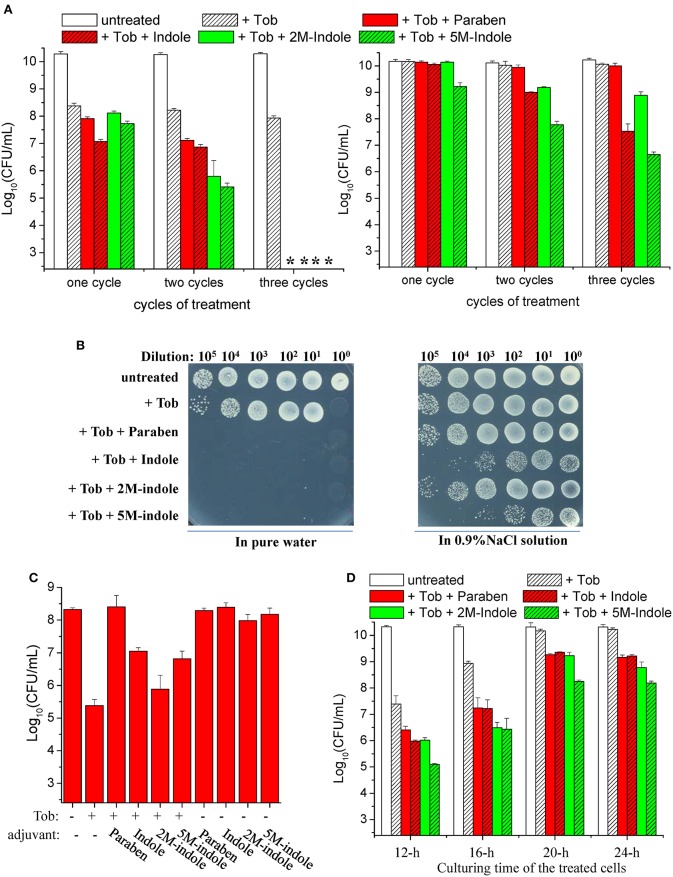
Indole derivatives potentiate tobramycin against stationary-phase *S. aureus*. **(A,B)** Survival of stationary-phase *S. aureus* following three cycles of 5-min treatment with 500 μg/ml tobramycin (Tob) plus 4 mM indicated adjuvant dissolved (left) in pure water or (right) in 0.9% NaCl solution. After centrifugation and complete removal of the supernatant, cell pellets were resuspended with the working solution and incubated for 5 min before spots were plated on LB agar dishes to count CFUs. **(A)** Quantitative results. The asterisk (*) means CFUs were undetectable. **(B)** Original cell survival results on dishes. Cycled treatment was performed by washing the treated cells with PBS once before the following round of treatment. *P* < 0.001 for the majority of the values indicated and *P* < 0.01 for 2M-indol. **(C,D)** Survival of **(C)** exponential-phase (OD_600_ ≈ 0.6) and **(D)** stationary-phase *S. aureus* cells following 5 min treatment with **(C)** 100 μg/ml or **(D)** 500 μg/ml Tob plus 4 mM indicated adjuvant dissolved in pure water. **(D)** Stationary-phase cells at different culturing time points were examined. Data represent the mean ± SD of three replicates, with independent experiments being performed at least three times.

We found that a 5-min combined treatment with tobramycin plus each adjuvant could eradicate stationary-phase *S. aureus* cells (left part in Figure 1A). In particular, no colony forming units (CFUs) were detected on LB dishes after the cells underwent the combined treatment three times (left part in Figure 1B), indicating that the number of viable cells was reduced by more than six orders of magnitude. In comparison, treatments with each adjuvant alone for three times did not kill the cells (Figure S1C). As expected, the presence of NaCl in the working solution significantly suppressed such tobramycin potentiation by paraben and indole (right parts in Figures 1A,B).

We also examined the effects of indole on other aminoglycoside antibiotics (streptomycin, gentamicin, and kanamycin). We found that they were hardly enhanced by indole, 2M-indole, 5M-indole, or paraben against *S. aureus* cells (Figure S2A). Similar observations were made with stationary-phase cells of other *S. aureus* strains, with gentamicin and kanamycin being slightly potentiated (Figures S2B, S2C). These results suggest that 5M-induced potentiation against *S. aureus* cells is largely specific to tobramycin. In addition, we examined β-lactams (mecillinam and meropenem) and fluoroquinolones (ofloxacin and ciprofloxacin), two types of commonly used bactericidal antibiotics. We found (i) that indole and 2M-indole potentiated ciprofloxacin, but not mecillinam, meropenem, and ofloxacin, by about one order of magnitude against stationary-phase *S. aureus* cells and (ii) that 5M-indole exhibited stronger potentiating effects, of around two orders of magnitude (Figure S2D). These observations suggest that 5M-indole-induced potentiation is largely specific to aminoglycosides.

Strikingly, we observed suppressive effects of each adjuvant on the action of tobramycin against exponential-phase *S. aureus* cells, i.e., they all suppressed the hypoionic shock-induced tobramycin potentiation to a certain degree (Figure 1C). Prompted by these observations, we examined the effects of these adjuvants on the action of tobramycin against stationary-phase *S. aureus* cells at different culturing time points. When the cells were cultured for 12, 16, 20, and 24 h and subsequently subjected to the combined treatment, the hypoionic shock-induced tobramycin potentiation was additively reduced, but each adjuvant was able to enhance the potentiation in each sample, with 5M-indole being the most potent (Figure 1D).

### 5M-Indole Potentiates Tobramycin Against Stationary-Phase *S. aureus* Cells in a Time- and Dose-Dependent Manner

**Figure 2:**
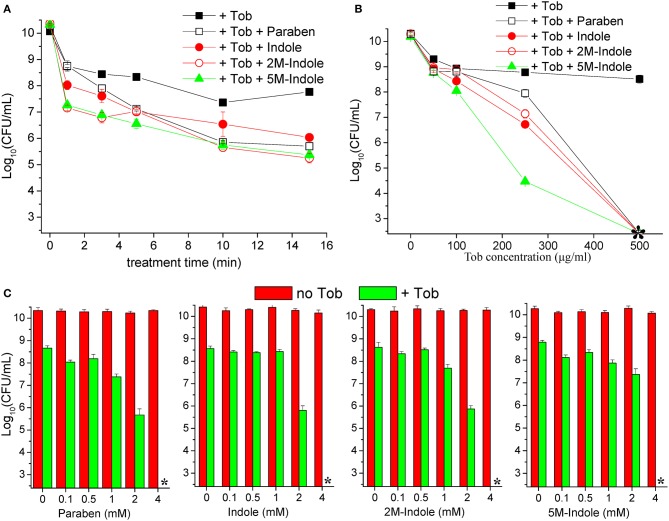
Indole derivatives potentiate tobramycin against stationary-phase *S. aureus* in a time- and dose-dependent manner. **(A)** Survival of stationary-phase *S. aureus* (from 16 h cultures) following treatment with 500 μg/ml tobramycin (Tob) plus 4 mM indicated adjuvant for varying lengths of time. **(B,C)** Survival of stationary-phase *S. aureus* following three cycles of 5-min treatment with **(B)** varying concentrations of Tob plus 4 mM indicated adjuvant or **(C)** 500 μg/ml Tob plus varying concentrations of adjuvant. Data represent the mean ± SD of three replicates, with independent experiments being performed at least three times. The asterisk (^*^) means CFUs were undetectable.

We next examined the time and dose dependency of the combined treatment. The stationary-phase *S. aureus* cells grown in LB medium for 16 h were subjected to the combined treatment for varying lengths of time. The cell survival assay revealed that 1- or 3-min combined treatment already reached a maximum killing efficiency (except for the 1-min combined treatment with paraben) (Figure 2A), and extension of the treatment from 3 to 15 min only resulted in a limited increase in the killing efficiency. This observation indicates that tobramycin potentiation by these adjuvants can be achieved within a couple of minutes. As expected, such potentiation depends on the concentration of tobramycin. Specifically, weak potentiation was observed when the tobramycin concentration was below 100 μg/ml, and significant potentiation was detected at tobramycin concentrations above 250 μg/ml (Figure 2B). When 250 μg/ml tobramycin was used, 5M-indole and paraben were again the most and the least potent, respectively. In particular, no CFUs were detected on LB dishes when the cells were treated with 500 μg/ml tobramycin plus 4 mM adjuvant (indicated by an asterisk in Figure 2B). Furthermore, each adjuvant was found to reinforce tobramycin in a concentration-dependent manner (Figure 2C).

### 5M-Indole Potentiates Tobramycin and Streptomycin Killing of Antibiotic-Tolerant *S. aureus* Persisters and MRSA Cells

Stationary-phase *S. aureus* cells, which are highly tolerant to bactericidal antibiotics (Keren et al., 2004), are usually considered as one type of *S. aureus* persisters (Allison et al., 2011; Wang et al., 2018). Therefore, our aforementioned observations suggested that 5M-indole enables aminoglycosides to kill *S. aureus* persisters. To confirm this, we tested other types of *S. aureus* persisters. We omitted paraben, because it is less potent than indole, 2M-indole, and 5M-indole (Figures 2A,B).

**Figure 3:**
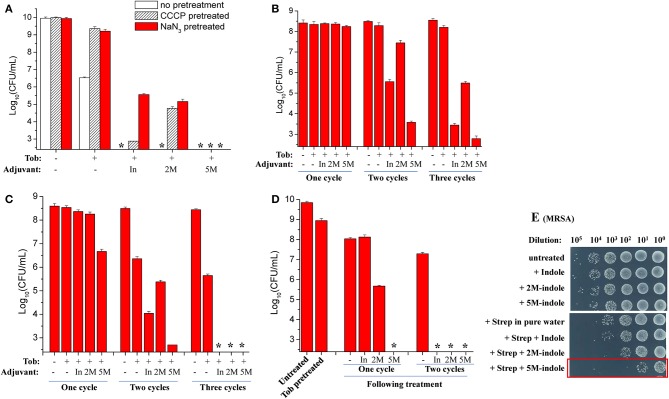
5M-indole potentiates tobramycin against *S. aureus* persisters and streptomycin against MRSA. **(A–D)** Survival of **(A)** ATP depletion-associated, **(B)** nutrient shift-induced, **(C)** starvation-induced, and **(D)** tobramycin (Tob)-tolerant *S. aureus* persister cells following cycled 5-min treatments with Tob plus 4 mM adjuvant (indole, 2M-indole, or 5M-indole) dissolved in pure water. In, indole; 2M, 2M-indole; 5M, 5M-indole. **(A)** Stationary-phase cells (from 12 h cultures) were pretreated with 100 μM CCCP or 15 μM NaN_3_ for 1 h prior to two cycles of treatments with 500 μg/ml Tob plus 4 mM adjuvant. **(B)** Exponential-phase cells (OD_600_ = 0.5–0.6) were transferred to fumarate containing M9 medium to prepare nutrient shift-induced persister cells, which were then subjected to cycled treatments with 100 μg/ml Tob plus 4 mM adjuvant. **(C)** Stationary-phase cells were transferred to yeast nitrogen broth medium without amino acids to prepare starvation-induced persister cells, which were then subjected to cycled treatments with 250 μg/ml Tob plus 4 mM adjuvant. **(D)** Stationary-phase cells were treated with 500 μg/ml Tob for 1 h under agitation at 37°C before being subjected to cycled treatments with 250 μg/ml Tob plus 4 mM adjuvant. For experimental details, refer to the Methods section. Data represent the mean ± SD of three replicates, with independent experiments being performed at least three times. The asterisk (^*^) means CFUs were undetectable. **(E)** Survival of stationary-phase MRSA cells following 5-min treatment with 7 mM adjuvant (indole, 2M-indole, or 5M-indole) plus 2,000 μg/ml streptomycin (Strep). MRSA cells (10 μl) were centrifuged and the cell pellets were resuspended in 100 μl working solution for treatment; MRSA cells without dilution were treated similarly (for the results see Figure S4A). It should be pointed out that 4 mM adjuvants exhibited little potentiating effect on Strep against MRSA cells (Figure S4B).

The ATP level is critical for bacterial persistence (Conlon et al., 2016; Shan et al., 2017; Pu et al., 2019). Therefore, we first examined ATP depletion-associated *S. aureus* persisters (Conlon et al., 2016) which were prepared by pretreating *S. aureus* cells with the protonophore CCCP for 1 h (Grassi et al., 2017). The cell survival assay revealed that CCCP pretreatment only exhibited marginal suppressive effects on indole- or 2M-indole-induced tobramycin potentiation and had little effect on 5M-indole-induced potentiation (Figure 3A). In line with these observations, NaN_3_ (an inhibitor of the electron transport chain), although it marginally suppressed indole- or 2M-indole-induced tobramycin potentiation, had no effects on 5M-indole-induced potentiation (Figure 3A). We confirmed that pretreatment with CCCP or NaN_3_ did significantly decrease the intracellular ATP level of *S. aureus* cells (Figure S3A). These results thus suggest 5M-indole-induced tobramycin potentiation could kill ATP depletion-associated *S. aureus* persisters, largely in a PMF-independent manner.

Second, we examined nutrient shift-induced *S. aureus* persisters by switching the carbon source of exponential-phase *S. aureus* cells to fumarate (Radzikowski et al., 2016). Such *S. aureus* persister cells exhibited high tolerance to tobramycin under conventional treatment conditions (Figure S3B). However, they could be effectively killed by two or three cycles of combined treatment with tobramycin plus indole or 5M-indole, with 2M-indole being less effective (Figure 3B).

Third, we examined starvation-induced *S. aureus* persisters by transferring stationary-phase *S. aureus* cells to medium without any nutrients (Chen et al., 2019). Such starvation-induced *S. aureus* persister cells were hardly killed by tobramycin under conventional treatment condition (Figure S3C). However, approximately 99% of the cells were killed by one cycle of combined treatment with tobramycin plus 5M-indole, and two or three cycles of treatments could further kill the persisters, with indole being the least potent (Figure 3C and Figure S3D).

Lastly, we directly examined antibiotic-tolerant cells, which were selected by agitating a stationary-phase *S. aureus* cell culture with supralethal (500 μg/ml) tobramycin concentrations for 1 h. The cell survival assay revealed that around 90% of cells present in the stationary-phase culture were tolerant to the 1 h tobramycin treatment, and that such tobramycin-tolerant persisters cells could be completely killed by the combined treatment with tobramycin plus 5M-indole (indicated by the asterisk in Figure 3D and Figure S3E), with indole and 2M-indole being less effective. One more cycle of the combined treatment with indole and 2M-indole completely eradicated the persisters (Figure 3D and Figure S3E). Collectively, these observations demonstrate that 5M-indole is highly potent in potentiating tobramycin to kill various types of antibiotic-tolerant *S. aureus* persister cells, with indole and 2M-indole being less effective.

In addition, we demonstrate that the combined treatment is able to kill methicillin-resistant *S. aureus* (MRSA), which is listed as one of most dangerous bacterial pathogens by the WHO (WHO, 2014, 2017). The cell survival assay showed that only three cycles of combined treatments with streptomycin plus 5M-indole exerted certain lethal effects on stationary-phase MRSA cells (red frame, Figure S4A). We then diluted the MRSA cells 10-fold before treating them once, and found that around 99.9% of the MRSA cells were killed by the combined treatment with streptomycin plus 5M-indole (red frame, Figure 3E), while only 90% of the cells were killed by streptomycin alone. It should be pointed out that the concentration of adjuvants used in this assay was 7 mM, and that 4 mM adjuvants exhibited no or limited potentiating effects (Figure S4B).

### 5M-Indole Potentiates Aminoglycosides Against Many Strains of Gram-Positive Bacteria but Not Gram-Negative Bacteria

**Figure 4:**
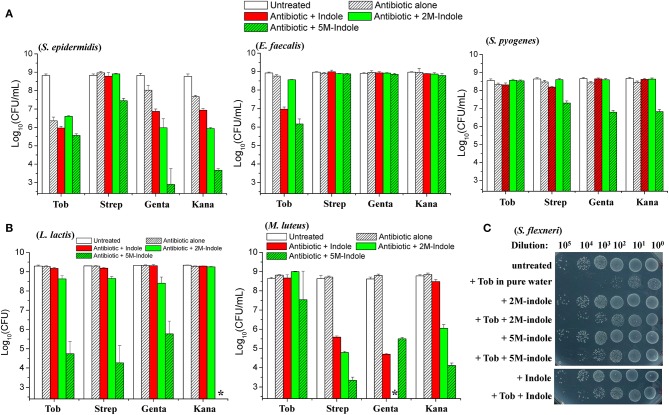
5M-indole potentiates aminoglycosides against many strains of gram-positive bacteria, including MRSA, but not gram-negative bacteria. **(A,B)** Survival of stationary-phase cells of indicated bacterial strains following 5-min treatment with 10 mM adjuvant (indole, 2M-indole, or 5M-indole) plus 500 μg/ml tobramycin (Tob), 2,000 μg/ml streptomycin (Strep), 500 μg/ml gentamycin (Genta), or 1,000 μg/ml kanamycin (Kana) dissolved in pure water. Data represent the mean ± SD of three replicates, with independent experiments being performed at least three times. **(C)** Survival of stationary-phase *S. flexneri* following 5-min treatment with 1 mM adjuvant plus 200 μg/ml Tob dissolved in pure water. Similar results for *E. coli* are shown in Figure S6E. The asterisk (*) means CFUs were undetectable.

We next examined whether indole could potentiate aminoglycoside antibiotics against other bacterial strains than *S. aureus*. When in the exponential-phase stage, these bacteria exhibit varying sensitivity to the aminoglycoside antibiotics tobramycin, streptomycin, gentamicin, and kanamycin (Figure S5). Regarding the gram-positive pathogens *Staphylococcus epidermidis, Enterococcus faecalis*, and *Streptococcus pyogenes*, we found that (i) indole, 2M-indole, and 5M-indole reinforced gentamicin and kanamycin against *S. epidermidis* (left part in Figure 4A); (ii) indole and 5M-indole potentiated tobramycin against *E. faecalis* (middle part in Figure 4A); and (iii) 5M-indole potentiated streptomycin, gentamicin, and kanamycin against *S. pyogenes* (right part in Figure 4A). We also examined two industrial, non-pathogenic gram-positive bacterial strains, *Micrococcus luteus* and *Lactococcus lactis*, and found that (i) all these aminoglycosides were potentiated by 5M-indole to kill *L. lactis* (left part of Figure 4B); and (ii) streptomycin, gentamicin, and kanamycin were potentiated by 2M-indole and 5M-indole to kill *M. luteus* (right part of Figure 4B). It should be pointed out that the concentration of adjuvants used in these assay is 10 mM, and that 4 mM adjuvants exhibited no (Figures S6A–S6C) or limited (Figure S6D) potentiating effects.

Lastly, we examined two gram-negative bacteria. Unexpectedly, indole, 2M-indole, and 5M-indole all effectively suppressed the bactericidal action of tobramycin against stationary-phase *Shigella flexneri* (Figure 4C) and *E. coli* (Figure S6E) under hypoionic condition. Prompted by these observations, we further examined exponential-phase *E. coli* cells and observed similar suppressive effects (Figure S6F). These observations on different bacterial strains, though not exhaustive, indicate that 5M-indole is able to enhance the hypoionic shock-induced tobramycin potentiation against gram-positive bacteria in the stationary phase, but suppresses the potentiating effect against gram-positive bacteria in the exponential phase and against gram-negative bacteria in both phases.

### 5M-Indole Potentiates Tobramycin Against *S. aureus* Persisters in Mice

**Figure 5:**
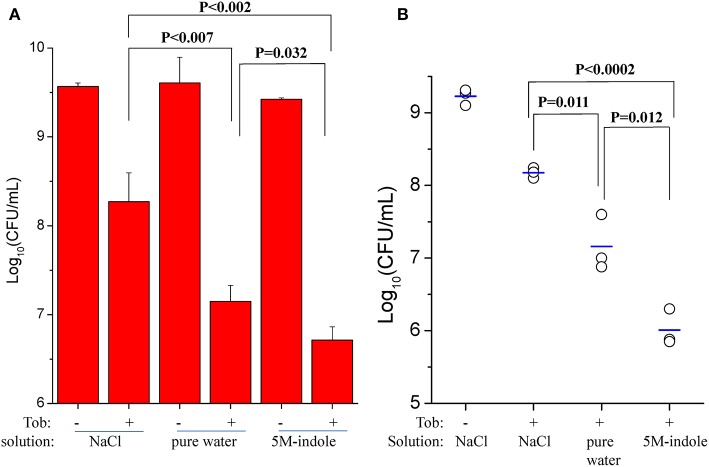
5M-indole potentiates tobramycin against *S. aureus* in mice. **(A,B)** Survival of stationary-phase *S. aureus* in **(A)** isolated mouse skin or **(B)** wounds in mice following 5-min treatment with 100 μg/ml tobramycin (Tob) and 5M-indole dissolved in NaCl solution or pure water. Data represent the mean ± SD of three replicates, with independent experiments being performed at least three times. For experimental details, please refer to the Methods section.

We also investigated whether 5M-indole is able to facilitate aminoglycoside antibiotics to eradicate *S. aureus* cells in animals. First, we removed skin from sacrificed mice and then placed stationary-phase *S. aureus* cells on the skin before subjecting them to the combined treatment. The cell survival assay showed that tobramycin dissolved in pure water killed the *S. aureus* cells more effectively than it did in 0.9% NaCl solution (*P* < 0.007, Figure 5A and Figure S7A), demonstrating the hypoionic shock-induced tobramycin potentiation under *ex vivo* conditions. More importantly, the presence of 5M-indole could further enhance tobramycin against *S. aureus* in comparison with tobramycin treatment alone (*P* = 0.035, Figure 5A).

Second, we utilized an acute skin wound model (Davidson, 1998) to measure the *in vivo* efficacy of the combined treatment. To this end, a piece of skin of 1 cm × 1 cm was removed from the right back of anesthetized mice (Figure S7B), and *S. aureus* cells were placed on the wound before subjecting the mice to the combined treatment. Initially, we analyzed stationary-phase *S. aureus* cells and observed a marginal potentiating effect of 5M-indole on tobramycin (Figure S7C). Then we analyzed ATP depletion-associated *S. aureus* persister cells as induced by CCCP pretreatment (Figure 3A), and found that 5M-indole significantly enhanced the bactericidal action of tobramycin (*P* = 0.012, Figure 5B and Figure S7D). Again, tobramycin dissolved in pure water killed the cells more much efficiently than it did in NaCl solution (*P* = 0.011, Figure 5B).

### Effective Abolishment of 5M-Indole-Induced Tobramycin Potentiation Against *S. aureus* by Cations and EDTA

We attempted to dissect the biochemical mechanisms underlying this unique 5M-indole-induced tobramycin potentiation against stationary-phase *S. aureus* cells. Considering that the absence of electrolyte is a prerequisite for the hypoionic shock-induced aminoglycoside potentiation, as we reported previously (Jiafeng et al., 2015), and the presence of 0.9% NaCl significantly suppressed 5M-indoled-induced tobramycin potentiation (right parts in Figures 1A,B), we systematically examined the effects of salts.

**Figure 6:**
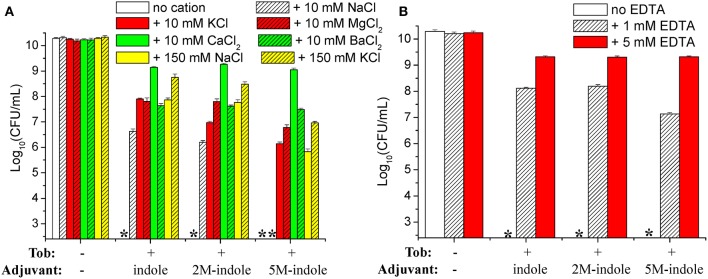
Salts and EDTA effectively abolish 5M-indole-induced tobramycin potentiation against *S. aureus*. **(A,B)** Survival of stationary-phase *S. aureus* following three cycles of 5-min treatment with 500 μg/ml tobramycin (Tob) plus 4 mM adjuvant (indole, 2M-indole, or 5M-indole) dissolved in pure water, in the absence or presence of **(A)** indicated salts or **(B)** EDTA. Data represent the mean ± SD of three independent experiments. The asterisk (^*^) means CFUs were undetectable.

The cell survival assay revealed that the presence of 10 mM KCl, MgCl_2_, CaCl_2_, or BaCl_2_ in the treatment solution effectively suppressed indole-, 2M-indole-, or 5M-indole-induced tobramycin potentiation against *S. aureus* cells, with CaCl_2_ being the most potent (Figure 6A). Notably, the presence of 10 mM NaCl significantly suppressed tobramycin potentiation by indole or 2M-indole, but not that by 5M-indole (indicated by the fourth asterisk in Figure 6A), and the presence of 150 mM NaCl or KCl significantly suppressed 5M-indole-induced tobramycin potentiation.

If the presence of MgCl_2_, CaCl_2_, or BaCl_2_ can suppress indole-induced tobramycin potentiation, can the absolute absence of them enhance such potentiation? We examined this question by treating the cells with solutions containing EDTA, a divalent cation chelator. Unexpectedly, the cell survival assay revealed that the presence of 1 mM or 5 mM EDTA almost abolished the tobramycin potentiation by indole, 2M-indole, and 5M-indole (Figure 6B). The mechanisms underlying this EDTA-mediated suppressive effect were not investigated in this study; we hypothesize it results from the disturbing effect of EDTA on the cell envelope of *S. aureus* cells (Felix, 1982; Hancock, 1984).

## Discussion

Several notable findings have been made in this study. First, indole derivatives are able to effectively and rapidly potentiate aminoglycoside killing of antibiotic-tolerant *S. aureus* persister cells, MRSA cells, and other gram-positive pathogens, with 5M-indole being the most potent. Second, 5M-indole-induced potentiation is largely independent of the PMF. Third, 5M-indole facilitates aminoglycoside to kill *S. aureus* in a mouse model. These findings may guide the development of indole derivatives as adjuvants for aminoglycoside potentiation against gram-positive pathogens.

Our approach differs from earlier reports in several aspects (Lee et al., 2010; Han et al., 2011; Vega et al., 2012, 2013; Hu et al., 2015; Kwan et al., 2015; Wang et al., 2019). First, 5M-induced aminoglycoside potentiation is achieved within a couple of minutes. In contrast, bacterial eradication by a combined treatment with indole and antibiotics under conventional conditions takes a couple of hours (Hu et al., 2015; Han et al., 2017; Wang et al., 2019). Second, salts are able to abolish the potentiating effect. In other words, the absence of ions is a prerequisite for such aminoglycoside potentiation. Third, the potentiating effect is largely independent of the PMF; the PMF is well-known to be indispensable for aminoglycoside actions under conventional treatment conditions (as reviewed in Taber et al., 1987) and also for metabolite-stimulated aminoglycoside potentiation (Allison et al., 2011; Barraud et al., 2013; Peng et al., 2015; Meylan et al., 2017; Su et al., 2018). In this regard, indole-induced aminoglycoside potentiation represents a unique mechanism. Notably, Conlon and colleagues recently reported that rhamnolipid-induced aminoglycoside potentiation was also independent of the PMF and only effective against gram-positive, but not gram-negative, pathogens (Radlinski et al., 2019), in agreement with our present findings. Nevertheless, this approach, as the metabolite-based approach, takes several hours to achieve a significant potentiating effect.

Indole, an endogenous metabolite in many bacteria, has multiple and diverse roles in signaling (Lee et al., 2010), and it even mediates the signaling between enteric bacteria and their mammalian host (Bansal et al., 2010). Opposite effects of indole on the antibiotic resistance of bacteria have been reported previously. For instance, indole was shown to increase (Lee et al., 2010; Han et al., 2011; Vega et al., 2012, 2013) or decrease (Hu et al., 2015; Kwan et al., 2015) the antibiotic tolerance of *E. coli*, depending on experimental conditions. In addition, indole was recently reported to dramatically reduce the antibiotic resistance of *L. enzymogenes* at the early exponential phase, but it exhibited little effect on the same bacterium in late exponential and stationary phases (Wang et al., 2019). In our study, indole derivatives appeared to potentiate aminoglycoside only against stationary-phase gram-positive bacteria under hypoionic conditions but they suppressed the effects of aminoglycosides against gram-positive bacteria in exponential-phase stage and gram-negative bacteria in both stages. These distinct or opposite effects are likely linked to different signaling pathways or biomolecules, which are activated or inhibited by indole under different experimental conditions and/or bacterial statuses. Considering that 5M-indole only effectively potentiates tobramycin, but not other aminoglycosides, against *S. aureus* cells, it seems that such biomolecules have a certain preference toward different aminoglycoside antibiotics (Figures S2A–S2C).

The precise mechanisms underlying indole-induced aminoglycoside potentiation remain unclear. We speculate that such potentiation is linked to the bacterial cell membrane based on the following considerations. First, the potentiating effect was only observed in gram-positive bacteria, but not in gram-negative bacteria, which are different mainly in their cell membranes. Second, indole has been shown to be able to act as an ionophore to dissipate the PMF across the cell membrane (Chimerel et al., 2012). In addition, we found that EDTA, which is well-known to disintegrate the bacterial cell membrane (Felix, 1982; Hancock, 1984), abolished the potentiating effect (Figure 6B). Lastly, aminoglycosides have been shown to kill bacteria by inducing membrane protein aggregation and cell membrane damage (Davis et al., 1986). If the molecular mechanisms underlying indole-induced aminoglycoside potentiation can be dissected, certain indole derivatives may be developed that are able to potentiate aminoglycoside as effectively as 5M-indole but are inhibited by salts to a lower extent. Studies of the precise underlying mechanisms are currently underway in our laboratory.

## Data Availability Statement

All datasets generated for this study are included in the article/Supplementary Material.

## Ethics Statement

The animal study was reviewed and approved by the Animal Ethical and Welfare Committee of Fujian Normal University.

## Author Contributions

XF designed the research. FS, MB, ZL, and BL performed the research. YG managed the project. XF and YW analyzed the data. XF wrote the paper.

### Conflict of Interest

The authors declare that the research was conducted in the absence of any commercial or financial relationships that could be construed as a potential conflict of interest.

## References

[B1] Allison K. R. Brynildsen M. P. Collins J. J. (2011). Metabolite-enabled eradication of bacterial persisters by aminoglycosides. Nature 473, 216–220. 10.1038/nature10069 21562562 PMC3145328

[B2] Balaban N. Q. Helaine S. Lewis K. Ackermann M. Aldridge B. Andersson D. I.. (2019). Definitions and guidelines for research on antibiotic persistence. Nat. Rev. Microbiol. 17, 441–448. 10.1038/s41579-019-0196-3 30980069 PMC7136161

[B3] Bansal T. Alaniz R. C. Wood T. K. Jayaraman A. (2010). The bacterial signal indole increases epithelial-cell tight-junction resistance and attenuates indicators of inflammation. Proc. Natl. Acad. Sci. U.S.A. 107, 228–233. 10.1073/pnas.0906112107 19966295 PMC2806735

[B4] Barraud N. Buson A. Jarolimek W. Rice S. A. (2013). Mannitol enhances antibiotic sensitivity of persister bacteria in Pseudomonas aeruginosa biofilms. PLoS ONE 8:e84220. 10.1371/journal.pone.0084220 24349568 PMC3862834

[B5] Chen Z. Gao Y. Lv B. Sun F. Yao W. Wang Y.. (2019). Hypoionic shock facilitates aminoglycoside killing of both nutrient shift- and starvation-induced bacterial persister cells by rapidly enhancing aminoglycoside uptake. Front. Microbiol. 10:2028. 10.3389/fmicb.2019.02028 31551965 PMC6743016

[B6] Chimerel C. Field C. M. Pinero-Fernandez S. Keyser U. F. Summers D. K. (2012). Indole prevents Escherichia coli cell division by modulating membrane potential. Biochim. Biophys. Acta 18, 1590–1594. 10.1016/j.bbamem.2012.02.022 PMC3793866 22387460

[B7] Conlon B. P. Rowe S. E. Gandt A. B. Nuxoll A. S. Donegan N. P. Zalis E. A. (2016). Persister formation in *Staphylococcus aureus* is associated with ATP depletion. Nat. Microbiol. 1:16051 10.1038/nmicrobiol.2016.51 PMC4932909 27572649

[B8] Davidson J. M. (1998). Animal models for wound repair. Arch. Dermatol. Res. 290, S1–S11. 10.1007/PL00007448 9710378

[B9] Davis B. D. Chen L. L. Tai P. C. (1986). Misread protein creates membrane channels: an essential step in the bactericidal action of aminoglycosides. Proc. Natl. Acad. Sci. U.S.A. 83, 6164–6168. 10.1073/pnas.83.16.6164 2426712 PMC386460

[B10] Eng R. H. Padberg F. T. Smith S. M. Tan E. N. Cherubin C. E. (1991). Bactericidal effects of antibiotics on slowly growing and nongrowing bacteria. Antimicrob. Agents Chemother. 35, 1824–1828. 10.1128/AAC.35.9.1824 1952852 PMC245275

[B11] Felix H. (1982). Permeabilized cells. Anal. Biochem. 120, 211–234. 10.1016/0003-269790340-2 6178313

[B12] Grassi L. Di Luca M. Maisetta G. Rinaldi A. C. Esin S. Trampuz A.. (2017). Generation of persister cells of pseudomonas aeruginosa and staphylococcus aureus by chemical treatment and evaluation of their susceptibility to membrane-targeting agents. Front. Microbiol. 8:1917. 10.3389/fmicb.2017.01917 29046671 PMC5632672

[B13] Han T. H. Lee J. H. Cho M. H. Wood T. K. Lee J. (2011). Environmental factors affecting indole production in *Escherichia coli*. Res. Microbiol. 162, 108–116. 10.1016/j.resmic.2010.11.005 21145393 PMC3171796

[B14] Han Y. Wang Y. Yu Y. Chen H. Shen Y. Du L. (2017). Indole-induced reversion of intrinsic multiantibiotic resistance in lysobacter enzymogenes. Appl. Environ. Microbiol. 83:e00995-17. 10.1128/AEM.00995-17 28625984 PMC5561299

[B15] Hancock R. E. (1984). Alterations in outer membrane permeability. Annu. Rev. Microbiol. 38, 237–264. 10.1146/annurev.mi.38.100184.001321 6093683

[B16] Hess D. J. Henry-Stanley M. J. Wells C. L. (2014). Antibacterial synergy of glycerol monolaurate and aminoglycosides in *Staphylococcus aureus* biofilms. Antimicrob. Agents. Chemother. 58, 6970–6973. 10.1128/AAC.03672-14 25182634 PMC4249351

[B17] Hirakawa H. Inazumi Y. Masaki T. Hirata T. Yamaguchi A. (2005). Indole induces the expression of multidrug exporter genes in Escherichia coli. Mol. Microbiol. 55, 1113–1126. 10.1111/j.1365-2958.2004.04449.x 15686558

[B18] Hoagland D. T. Liu J. Lee R. B. Lee R. E. (2016). New agents for the treatment of drug-resistant Mycobacterium tuberculosis. Adv. Drug Deliv. Rev. 102, 55–72. 10.1016/j.addr.2016.04.026 27151308 PMC4903924

[B19] Hu Y. Kwan B. W. Osbourne D. O. Benedik M. J. Wood T. K. (2015). Toxin YafQ increases persister cell formation by reducing indole signaling. Environ. Microbiol. 17, 1275–1285. 10.1111/1462-2920.12567 25041421

[B20] Jiafeng L. Fu X. Chang Z. (2015). Hypoionic shock treatment enables aminoglycosides antibiotics to eradicate bacterial persisters. Sci. Rep. 5:14247. 10.1038/srep14247 26435063 PMC4593029

[B21] Keren I. Kaldalu N. Spoering A. Wang Y. Lewis K. (2004). Persister cells and tolerance to antimicrobials. FEMS Microbiol. Lett. 230, 13–18. 10.1016/S0378-109700856-5 14734160

[B22] Kwan B. W. Osbourne D. O. Hu Y. Benedik M. J. Wood T. K. (2015). Phosphodiesterase DosP increases persistence by reducing cAMP which reduces the signal indole. Biotechnol. Bioeng. 112, 588–600. 10.1002/bit.25456 25219496

[B23] Lee H. H. Molla M. N. Cantor C. R. Collins J. J. (2010). Bacterial charity work leads to population-wide resistance. Nature 467, 82–85. 10.1038/nature09354 20811456 PMC2936489

[B24] Lee J. H. Kim Y. G. Gwon G. Wood T. K. Lee J. (2016). Halogenated indoles eradicate bacterial persister cells and biofilms. AMB Exp. 6:123. 10.1186/s13568-016-0297-6 27921270 PMC5138170

[B25] Lee J. H. Lee J. (2010). Indole as an intercellular signal in microbial communities. FEMS Microbiol. Rev. 34, 426–444. 10.1111/j.1574-6976.2009.00204.x 20070374

[B26] Lewis K. (2010). Persister cells. Annu. Rev. Microbiol. 64, 357–372. 10.1146/annurev.micro.112408.134306 20528688

[B27] Meylan S. Porter C. B. M. Yang J. H. Belenky P. Gutierrez A. Lobritz M. A.. (2017). Carbon sources tune antibiotic susceptibility in *Pseudomonas aeruginosa* via tricarboxylic acid cycle control. Cell. Chem. Biol. 24, 195–206. 10.1016/j.chembiol.2016.12.015 28111098 PMC5426816

[B28] Mitchell G. Lafrance M. Boulanger S. Seguin D. L. Guay I. Gattuso M.. (2012). Tomatidine acts in synergy with aminoglycoside antibiotics against multiresistant *Staphylococcus aureus* and prevents virulence gene expression. J. Antimicrob. Chemother. 67, 559–568. 10.1093/jac/dkr510 22129590

[B29] Moreau-Marquis S. O'Toole G. A. Stanton B. A. (2009). Tobramycin and FDA-approved iron chelators eliminate *Pseudomonas aeruginosa* biofilms on cystic fibrosis cells. Am. J. Respir. Cell. Mol. Biol. 41, 305–313. 10.1165/rcmb.2008-0299OC 19168700 PMC2742750

[B30] Nohr-Meldgaard K. Ovsepian A. Ingmer H. Vestergaard M. (2018). Resveratrol enhances the efficacy of aminoglycosides against *Staphylococcus aureus*. Int. J. Antimicrob. Agents 52, 390–396. 10.1016/j.ijantimicag.2018.06.005 29906565

[B31] O'Neill J. (2016). Tackling Drug-Resistant Infections Globally. Final Report and Recommendations. The Review on Antimicrobial Resistance. Available online at: https://amr-review.org/sites/default/files/160525_Final%20paper_with%20cover.pdf

[B32] Peng B. Su Y. B. Li H. Han Y. Guo C. Tian Y. M.. (2015). Exogenous alanine and/or glucose plus kanamycin kills antibiotic-resistant bacteria. Cell. Metab. 21, 249–261. 10.1016/j.cmet.2015.01.008 25651179

[B33] Pu Y. Li Y. Jin X. Tian T. Ma Q. Zhao Z.. (2019). ATP-dependent dynamic protein aggregation regulates bacterial dormancy depth critical for antibiotic tolerance. Mol. Cell. 73, 143–156.e144. 10.1016/j.molcel.2018.10.022 30472191

[B34] Radlinski L. C. Rowe S. E. Brzozowski R. Wilkinson A. D. Huang R. Eswara P.. (2019). Chemical induction of aminoglycoside uptake overcomes antibiotic tolerance and resistance in staphylococcus aureus. Cell. Chem. Biol. 26, 1355–1364. 10.1016/j.chembiol.2019.07.009 31402316 PMC6800641

[B35] Radzikowski J. L. Vedelaar S. Siegel D. Ortega A. D. Schmidt A. Heinemann M. (2016). Bacterial persistence is an active sigmaS stress response to metabolic flux limitation. Mol. Syst. Biol. 12:882. 10.15252/msb.20166998 27655400 PMC5043093

[B36] Shan Y. Brown Gandt A. Rowe S. E. Deisinger J. P. Conlon B. P. Lewis K. (2017). ATP-Dependent persister formation in escherichia coli. MBio 8:e02267-16. 10.1128/mBio.02267-16 28174313 PMC5296605

[B37] Su Y. B. Peng B. Li H. Cheng Z. X. Zhang T. T. Zhu J. X.. (2018). Pyruvate cycle increases aminoglycoside efficacy and provides respiratory energy in bacteria. Proc. Natl. Acad. Sci. U.S.A. 115, E1578–E1587. 10.1073/pnas.1714645115 29382755 PMC5816162

[B38] Taber H. W. Mueller J. P. Miller P. F. Arrow A. S. (1987). Bacterial uptake of aminoglycoside antibiotics. Microbiol. Rev. 51, 439–457. 10.1128/MMBR.51.4.439-457.1987 3325794 PMC373126

[B39] Trusts T. P. C. (2016). A Scientific Roadmap for Antibiotic Discovery. The Pew Charitable Trusts.

[B40] Vega N. M. Allison K. R. Khalil A. S. Collins J. J. (2012). Signaling-mediated bacterial persister formation. Nat. Chem. Biol. 8, 431–433. 10.1038/nchembio.915 22426114 PMC3329571

[B41] Vega N. M. Allison K. R. Samuels A. N. Klempner M. S. Collins J. J. (2013). Salmonella typhimurium intercepts Escherichia coli signaling to enhance antibiotic tolerance. Proc. Natl. Acad. Sci. U.S.A. 110, 14420–14425. 10.1073/pnas.1308085110 23946425 PMC3761632

[B42] Wang Y. Bojer M. S. George S. E. Wang Z. Jensen P. R. Wolz C.. (2018). Inactivation of TCA cycle enhances *Staphylococcus aureus* persister cell formation in stationary phase. Sci. Rep. 8:10849. 10.1038/s41598-018-29123-0 30022089 PMC6052003

[B43] Wang Y. Tian T. Zhang J. Jin X. Yue H. Zhang X. H.. (2019). Indole reverses intrinsic antibiotic resistance by activating a novel dual-function importer. MBio 10:e00676-19. 10.1128/mBio.00676-19 31138746 PMC6538783

[B44] WHO (2014). Antimicrobial Resistance GLOBAL Report on Surveillance.

[B45] WHO (2017). Global Priority List of Antibiotic-Resistant Bacteria to Guide Research, Discovery, and Development of New Antibiotics.

[B46] Yu Q. Griffin E. F. Moreau-Marquis S. Schwartzman J. D. Stanton B. A. O'Toole G. A. (2012). *In vitro* evaluation of tobramycin and aztreonam versus *Pseudomonas aeruginosa* biofilms on cystic fibrosis-derived human airway epithelial cells. J. Antimicrob. Chemother. 67, 2673–2681. 10.1093/jac/dks296 22843834 PMC3468082

