# Supplementary material for: 5-Methylindole Potentiates Aminoglycoside Against Gram-Positive Bacteria Including Staphylococcus aureus Persisters Under Hypoionic Conditions
Source: Front Cell Infect Microbiol. 2020 Feb 28;10:84. doi: 10.3389/fcimb.2020.00084 (PMC7058972; doi:10.3389/fcimb.2020.00084)
Supplement: Supplementary file 1 [file Data_Sheet_1.PDF]

Supporting information file

5-Methylindole Potentiates Aminoglycoside against Gram-positive Bacteria Including *Staphylococcus aureus* Persisters under Hypoionic Conditions

Fengqi Sun, Mengmeng Bian, Zhongyan Li, Boyan Lv, Yuanyuan Gao, Yan Wang and Xinmiao Fu

Table S1 Bacterial strains used in this study

Bacterial strains	Origins	Characteristics
Escherichia coli BW25113	the Nara Institute of Science and Technology (Ikoma, Nara, Japan)	G^-, Keio collection
Shigella flexneri 24T7T	A gift from Prof. Xiaoyun Liu at Peking University	G^-, Streptomycin-resistant
Staphylococcus aureus ATCC25923	A gift from Prof. Luhua Lai at Peking University	G^+
Staphylococcus aureus ATCC6538	ShangHai Luwei Microbial SCI&Tech Co, Ltd	G^+
Staphylococcus aureus CMCC(B)26003	The same as above	G^+
Methicillin-resistant *Staphylococcus aureus* ATCC43300	Hangzhou Binhe Microorganism Reagent Co, Ltd.	G^+, multi-drug resistant, streptomycin-sensitive
Staphylococcus epidermidis CMCC26069	The same as above	G^+, Streptomycin-resistant
Enterococcus faecalis ATCC29212	The same as above	G^+, multi-drug resistant
Streptococcus pyogenes ATCC19615	The same as above	G^+, multi-drug resistant
Micrococcus luteus CMCC28001	A gift from Dr. Qingeng Huang at Fujian Normal University	G^+, tobramycin- and kanamycin-resistant
Lactococcus lactis NZ9000	The same as above	G^+, multi-drug resistant

Table S2 Antibiotics, reagents and their concentrations used in this study

Antibiotics	Suppliers	Concentrations in use (μg/ml)
Tobramycin	Sangon Biotech (Shanghai) Co., Ltd.	Exponential-phase *S. aureus*: 100 μg/ml;<br>Stationary-phase *S. aureus*: 250, 500 μg/ml.<br>other gram-positive bacteria: 500 μg/ml.<br>*E. coli* and *S. flexneri*: 200 μg/ml.<br>Antibiotic sensitivity test: 25 μg/ml
Kanamycin	The same as above	Stationary-phase bacteria：1000 μg/ml<br>Antibiotic sensitivity test: 50 μg/ml
Gentamicin	The same as above	Stationary-phase bacteria：500 μg/ml<br>Antibiotic sensitivity test: 25 μg/ml
Streptomycin [c]	Beijing Solarbio Science & Technology Co., Ltd	Stationary-phase bacteria：2000 μg/ml<br>Antibiotic sensitivity test: 100 μg/ml
Meropenem	Dalian Meilun Biotechnology Co., LTD, China	Stationary-phase *S. aureus*: 20, 40, 60, 100, 200
Mecillinam	The same as above	Stationary-phase *S. aureus*: 20, 40, 60, 100, 200
Ofloxacin	Beijing Solarbio Science & Technology Co., Ltd.	Stationary-phase *S. aureus*: 5, 20, 40, 60, 100
Ciprofloxacin	Sangon Biotech (Shanghai) Co., Ltd.	Stationary-phase *S. aureus*: 5, 20, 40, 60, 100
CCCP	The same as above	100 μM
Indole	Aladdin Industrial Corporation Co., Ltd.	*S. aureus*: 4 mM; MRSA: 7 mM.<br>Other gram-positive bacteria: 10 mM<br>gram-negative bacteria: 1 mM
2-Methylindole	Sangon Biotech (Shanghai) Co., Ltd.	The same as above
5-Methylindole	The same as above	The same as above
Paraben	Aladdin Industrial Corporation Co., Ltd.	*S. aureus*: 4 mM

Figure S1

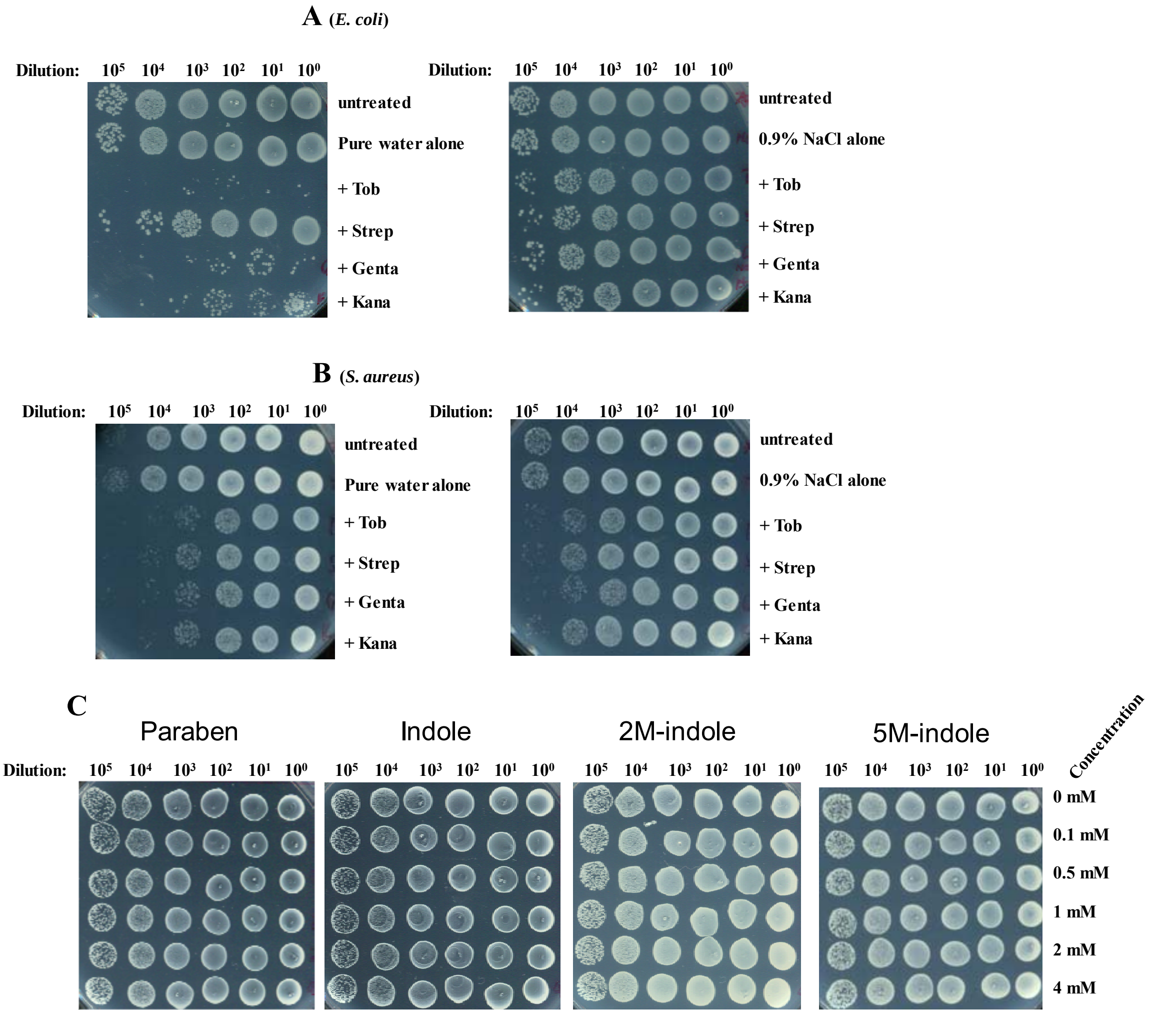

Fig. S1 Aminoglycosides exhibit limited killing effect against *S. aureus* stationary-phase cells under hypoionic conditions

(**A, B**) Survival of stationary-phase cells of *E. coli* (pane A) and *S. aureus* (panel B) following 5-min treatment with indicated concentrations of each aminoglycoside antibiotic as dissolved in pure water or in NaCl solution at concentrations as described in **Table S2**. Tob: tobramycin; Strep: streptomycin; Genta, gentamicin; Kana, kanamycin; (**C**) Survival of *S. aureus* stationary-phase cells following three cycles of treatment with paraben, indole, 2M-indole or 5M-indole at indicated concentrations.

Figure S2

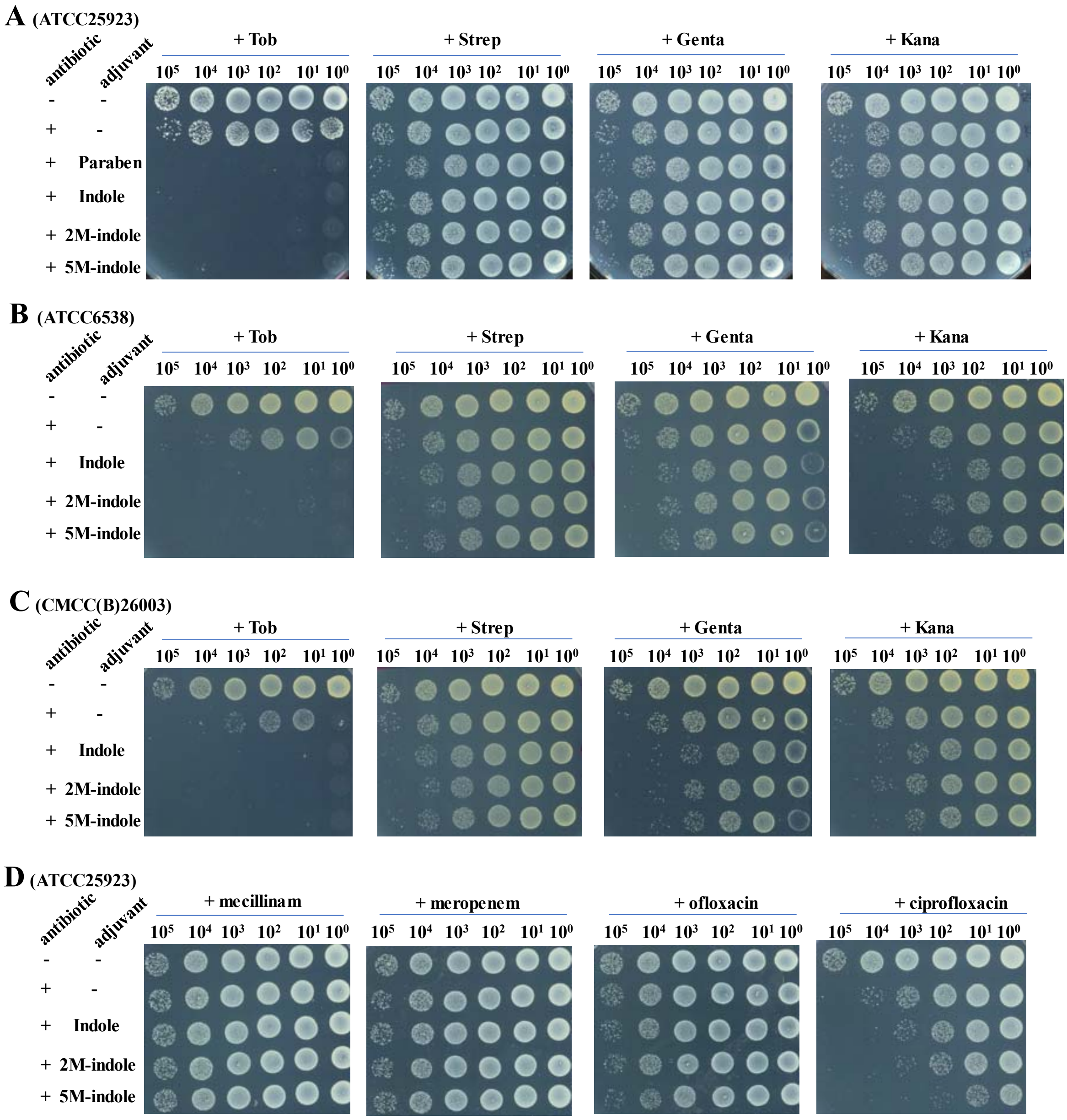

Fig. S2 5M-indole slightly potentiates other types of antibiotics against *S. aureus* cells

(**A**) Survival of stationary-phase cells of *S. aureus* ATCC25923 strain following three cycles of 5-min treatment with 4 mM adjuvant (indole, 2M-indole or 5M-indole) plus 500 μg/ml Tob, 2000 μg/ml Strep, 500 μg/ml Genta or 1000 μg/ml Kana dissolved in pure water. (**B**, **C**) Survival of stationary-phase cells of *S. aureus* ATCC6538 (panel B) and CMCC(B)26003 (panel C) strains following three cycles of 5-min treatment with 4 mM adjuvant (indole, 2M-indole or 5M-indole) plus 250 μg/ml Tob, 2000 μg/ml Strep, 250 μg/ml Genta or 500 μg/ml Kana dissolved in pure water. (**D**) Survival of *S. aureus* stationary-phase following three cycles of 5-min treatment with 160 μg/ml, 400 μg/ml meropenem, 100 μg/ml ofloxacin or 100 μg/ml ciprofloxacin plus 4 mM indicated adjuvant as dissolved in pure water. Cycled treatment was performed by washing the treated cells with PBS once and then subjected to the following round of treatment.

Figure S3

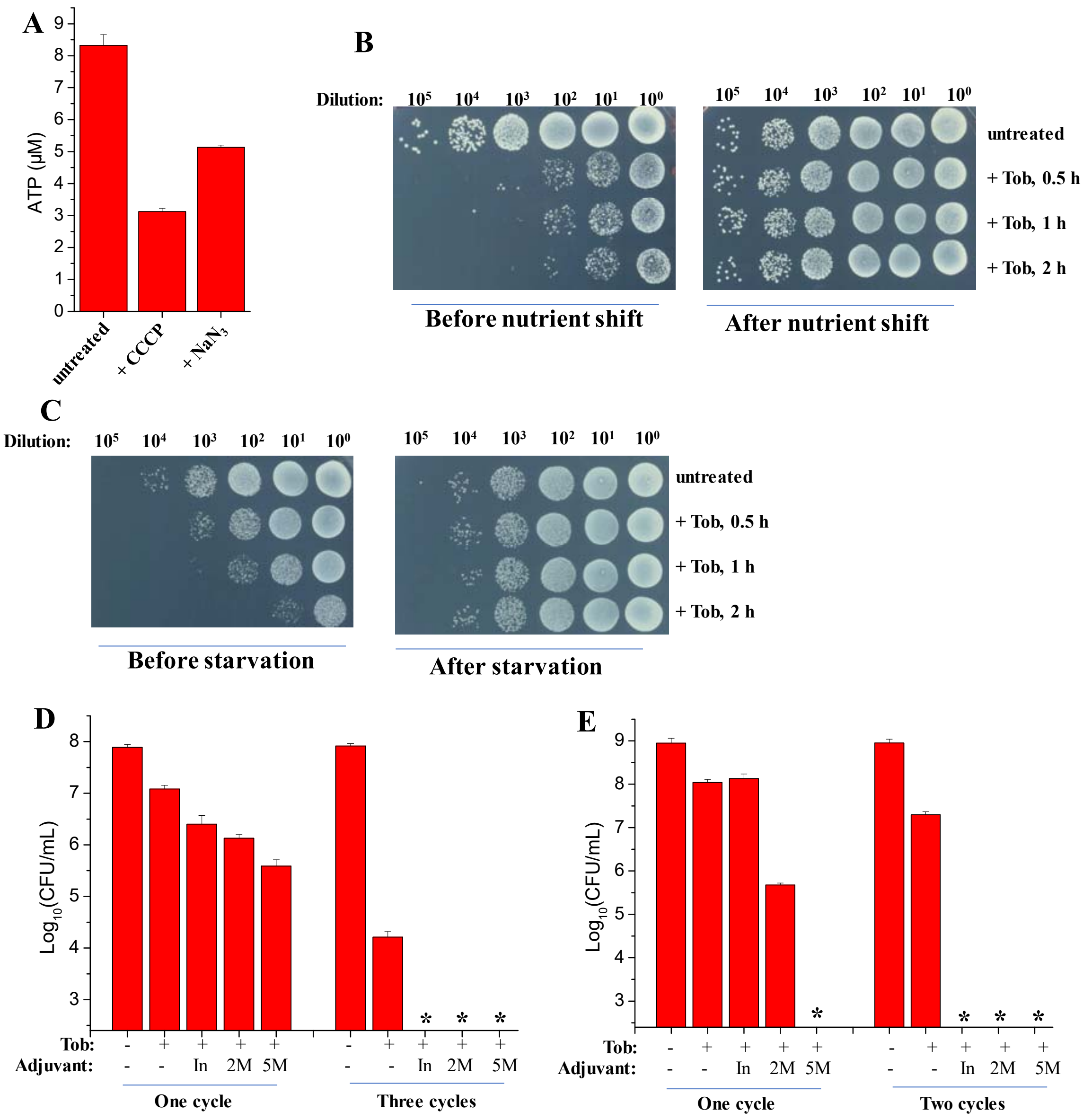

Fig. S3 Preparation of *S. aureus* persisters and their eradication by the combined treatment with tobramycin plus 5M-indole

(**A**) ATP levels in *S. aureus* stationary-phase cells after CCCP or NaN_3 treatment. Data represent the means ± SD of three replicates. (**B**) Survival of exponential-phase *S. aureus* cells before and after nutrient shift to fumarate following treatments with 100 μg/mL tobramycin for varying length of time. (**C**) Survival of stationary-phase *S. aureus* cells before and after starvation adaptation following treatments with 250 μg/mL tobramycin for varying length of time. For detail, refer to Methods section. (**D**, **E**) Survival of starvation-induced (panel D) and tobramycin-tolerant (panel E) *S. aureus* persister cells following cycled 5-min treatments with 500 μg/mL tobramycin plus 4 mM adjuvant (indole, 2M-indole or 5M-indole) dissolved in pure water.

Figure S4

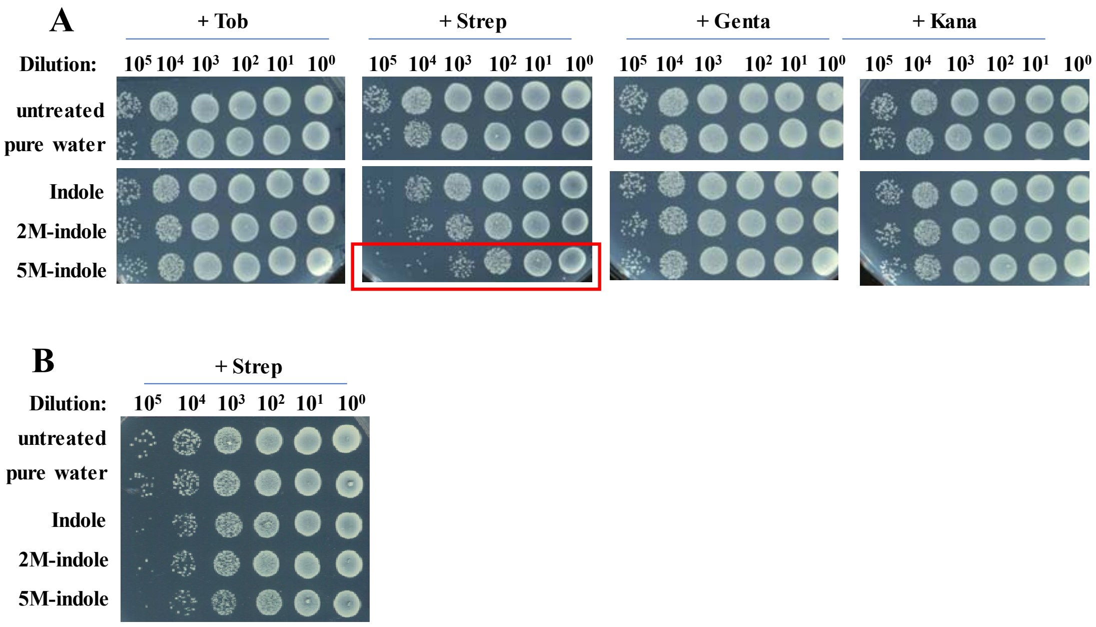

Fig. S4 Eradication of MRSA cells by the combined treatment with streptomycin plus 5M-indole

(**A**) Survival of stationary-phase MRSA cells following 5-min treatment with 7 mM adjuvant (indole, 2M-indole or 5M-indole) plus 500 μg/ml Tob, 2000 μg/ml Strep, 500 μg/ml Genta or 1000 μg/ml Kana dissolved in pure water. Red frame indicates the potentiating effect of 5M-indole on Strep. (**B**) Survival of stationary-phase MRSA cells following 5-min treatment with 4 mM adjuvant (indole, 2M-indole or 5M-indole) plus 2000 μg/ml Strep dissolved in pure water.

Figure S5

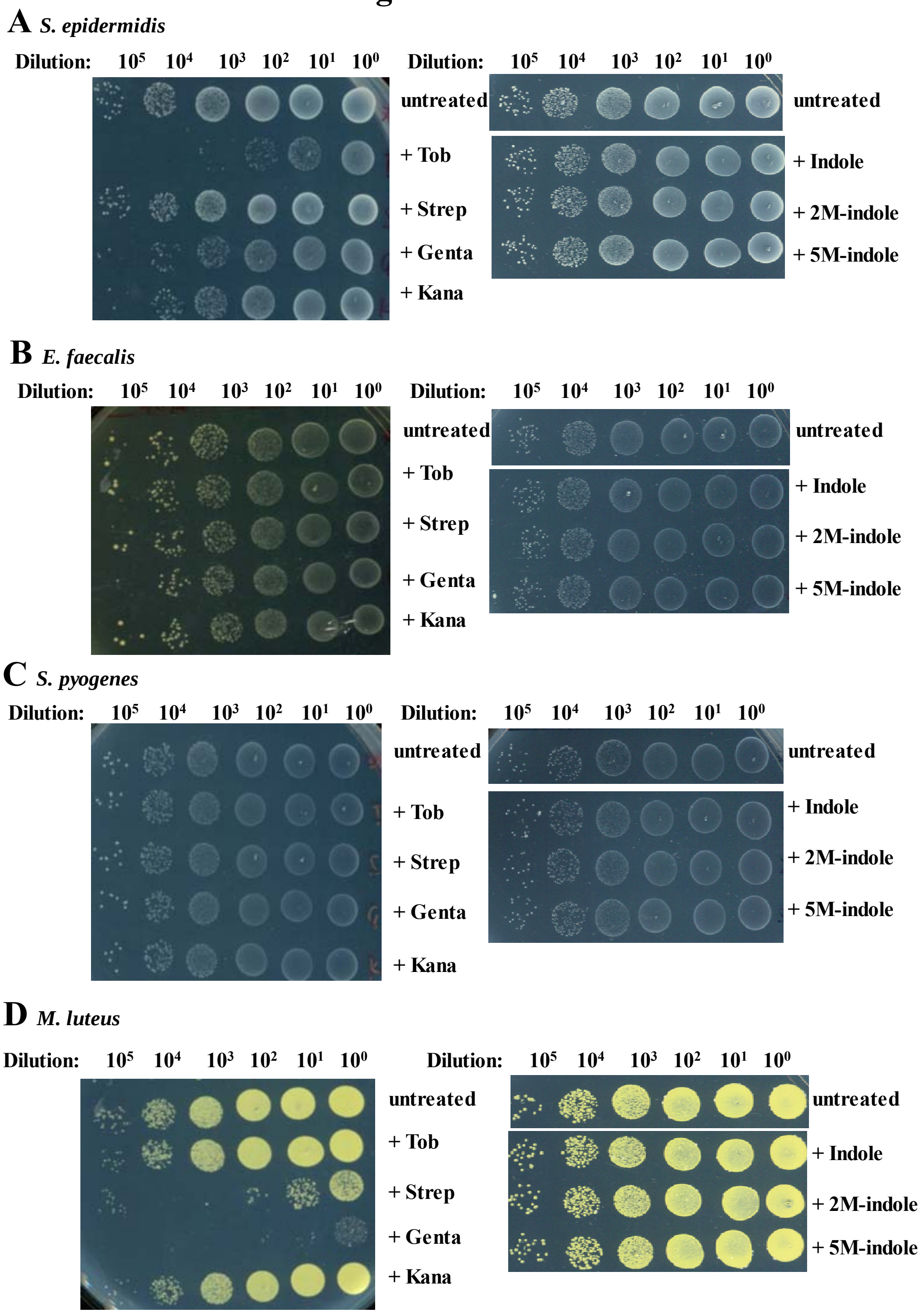

Fig. S5 Sensitivity of gram-positive bacteria to aminoglycosides and adjuvants.

(**A-D**) Left parts: survival of indicated bacterial strains in exponential-phase stage following treatments with each aminoglycoside antibiotic at concentrations as described in **Table S2** under conventional conditions (i.e., agitation at 37°C for 2 hours). Right parts: survival of indicated bacterial strains in stationary-phase stage following 5-min treatment with 10 mM indicated adjuvant.

Figure S6

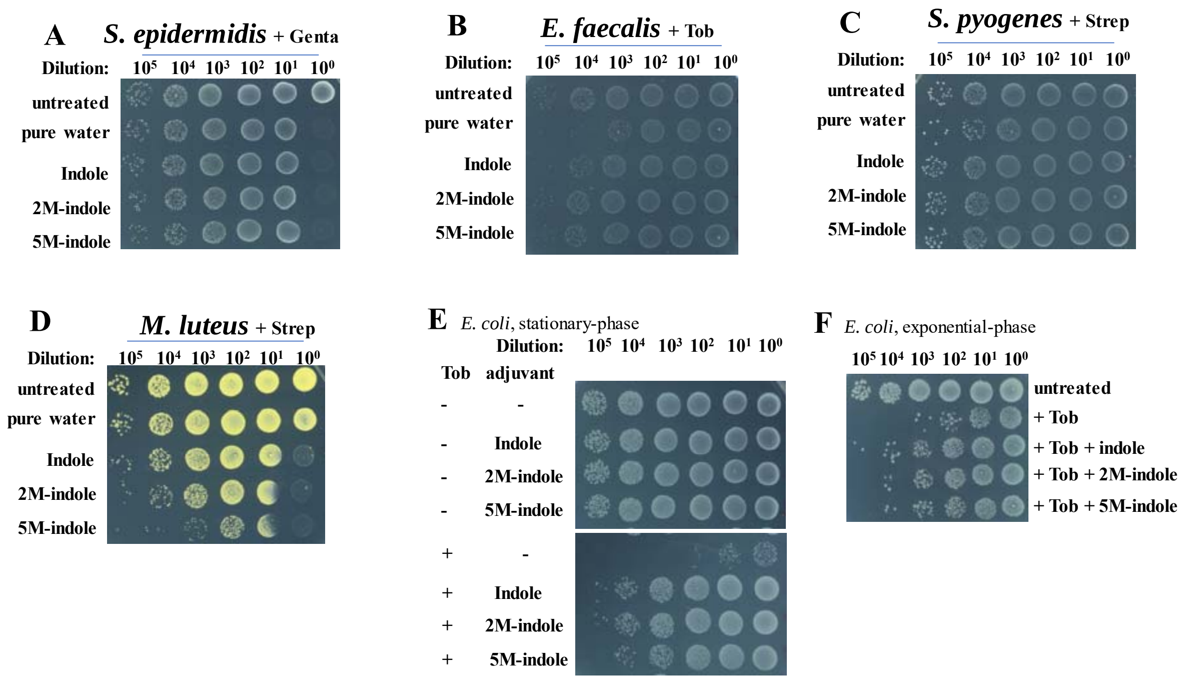

Fig. S6 5M-indole at low level hardly potentiates aminoglycosides against gram-positive bacteria and suppresses the action of tobramycin against *E. coli* cells

(**A**-**D**) Survival of stationary-phase cells of indicated bacterial strains following 5-min treatment with 4 mM adjuvant (indole, 2M-indole or 5M-indole) plus 500 μg/ml Tob, 2000 μg/ml Strep, 500 μg/ml Genta or 1000 μg/ml Kana dissolved in pure water. (**E**) Survival of *E. coli* stationary-phase cells following 5-min treatment with 1 mM adjuvant plus 200 μg/ml Tob dissolved in pure water. (**F**) Survival of *E. coli* exponential-phase cells following 5-min treatment with 1 mM adjuvant plus 25 μg/ml Tob dissolved in pure water.

Figure S7

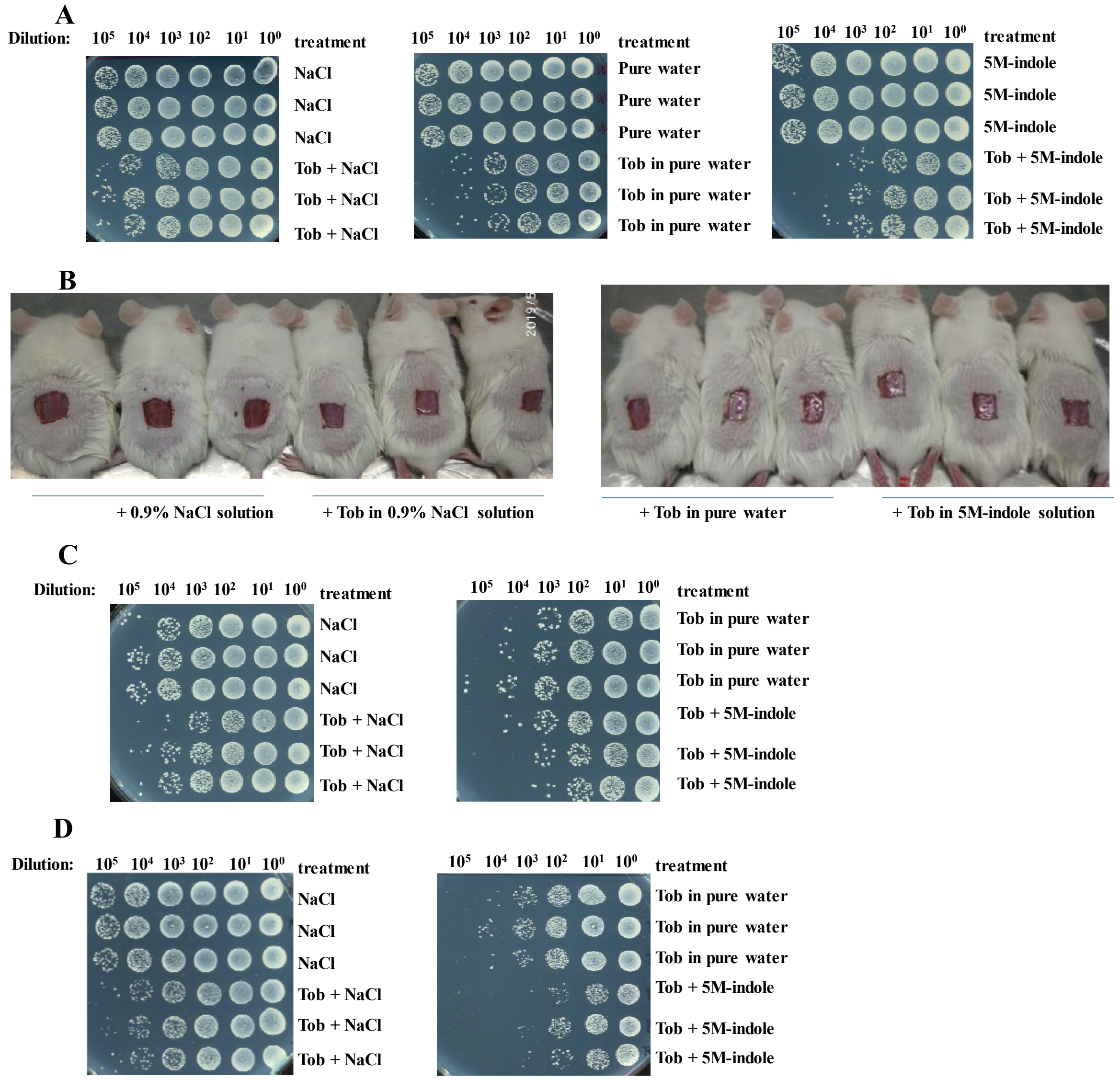

Fig. S7 5M-indole potentiates tobramycin against *S. aureus* cells *in vivo*

(**A**) Survival of stationary-phase *S. aureus* cells in the isolated mouse skin following 5-min treatment with 100 μg/mL Tob dissolved in pure water, NaCl- or 5M-indole-containing solution. Cells were centrifuged, re-suspended in pure water, plated on the isolated mouse skin and treated with the working solution. (**B**) Mice of an acute skin wound model, with a 1cm×1cm whole skin section being removed. **(C, D)** Survival of stationary-phase *S. aureus* cells in the wound of mice following 5-min treatment with 100 μg/mL Tob dissolved in pure water, NaCl- or 5M-indole-containing solution. Panel C: cells were centrifuged, re-suspended in pure water, plated on the wound of mice and treated twice with the working solution; after treatment, the muscles of wound were cut and homogenized, with the lysates being plated on LB dishes for bacterial survival assay. Panel D: cells were pretreated with 100 μM CCCP for one prior to the same treatment as described in Panel C.
